# Peripheral and respiratory muscle impairment during murine acute lung injury

**DOI:** 10.14814/phy2.15449

**Published:** 2022-09-06

**Authors:** Martín Angulo, Agustina Vacca, Romina Rodríguez, María Noel Marin, Ana Laura Suárez, Gissel Jorge, Oscar Nosiglia, Victoria Cambón, Anaclara Ríos, Matías Iglesias, Mariana Seija, Carlos Escande, Javier Hurtado, Arturo Briva

**Affiliations:** ^1^ Departamento de Fisiopatología, Hospital de Clínicas, Facultad de Medicina Universidad de la República Montevideo Uruguay; ^2^ Laboratorio de Exploración Funcional Respiratoria, Centro de Tratamiento Intensivo, Hospital de Clínicas, Facultad de Medicina Universidad de la República Montevideo Uruguay; ^3^ Laboratorio de Patologías del Metabolismo y Envejecimiento Institut Pasteur Montevideo Uruguay; ^4^ Unidad de Medicina Intensiva Hospital Español Montevideo Uruguay; ^5^ Cátedra de Medicina Intensiva, Hospital de Clínicas, Facultad de Medicina Universidad de la República Montevideo Uruguay

**Keywords:** acute lung injury, muscle atrophy, muscle dysfunction, muscle weakness

## Abstract

Acute respiratory distress syndrome is associated with skeletal muscle compromise, which decreases survival and impairs functional capacity. A comparative analysis of peripheral and respiratory muscles' atrophy and dysfunction in acute lung injury (ALI) has not been performed. We aimed to evaluate diaphragmatic and peripheral muscle mass and contractility in an ALI animal model. ALI was induced in C57BL/6 mice by intratracheal lipopolysaccharides instillation. Muscle mass and in vitro contractility were evaluated at different time points in hindlimb soleus (slow‐twitch) and extensor digitorum longus (EDL, fast‐twitch), as well as in the main respiratory muscle diaphragm. Myogenic precursor satellite cell‐specific transcription factor Pax7 expression was determined by Western blot. Lung injury was associated with atrophy of the three studied muscles, although it was more pronounced and persistent in the diaphragm. Specific contractility was reduced during lung injury in EDL muscle but restored by the time lung injury has resolved. Specific force was not affected in soleus and diaphragm. A persistent increase in Pax7 expression was detected in diaphragm and EDL muscles after induction of ALI, but not in soleus muscle. Different peripheral and respiratory skeletal muscles are distinctly affected during the course of ALI. Each of the studied muscles presented a unique pattern in terms of atrophy development, contractile dysfunction and Pax7 expression.

## Graphical abstract

Acute lung injury causes peripheral and respiratory muscle atrophy. Specific force was exclusively impaired in fast‐twitch hindlimb muscle. Pax7 expression increased only in hindlimb fast‐twitch muscle and diaphragm.

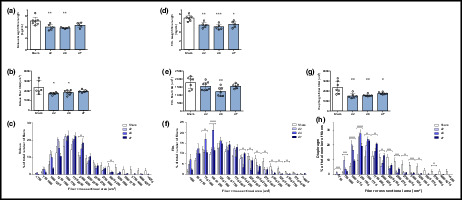

## 1 INTRODUCTION

Acute respiratory distress syndrome (ARDS) is a major cause of severe respiratory failure, frequently affecting patients with critical conditions like sepsis or trauma (Bellani et al., 2016; Pham & Rubenfeld, 2017). Many patients with ARDS develop skeletal muscle weakness, which typically persists for years after the resolution of lung injury (Fan et al., 2014). Muscle dysfunction is associated with increased long‐term mortality, reduced functional status, and worse quality of life (Dinglas et al., 2017; Fan et al., 2014; Herridge et al., 2011).

Different profiles of peripheral and respiratory muscle atrophy and weakness have been described in critically ill patients (Carambula et al., 2021; Dres et al., 2017; Puthucheary et al., 2013). Moreover, diverse muscle groups are affected to different degrees (Campbell et al., 1995; Jung et al., 2014). In humans with chronic respiratory diseases, for example, the respiratory muscles are differentially affected from the limb muscles (Barreiro & Gea, 2015). Recent studies using lung injury animal models have provided some insight into the mechanisms involved in skeletal muscle dysfunction (Chacon‐Cabrera et al., 2014; Files et al., 2012; Files et al., 2016; Marin‐Corral et al., 2010; Shieh et al., 2019). However, comprehensive analyses of ARDS‐related muscle atrophy and contractile dysfunction in different muscle groups and their progression patterns have not been performed. Most studies focus on either limb or respiratory muscles compromise, making it difficult to conclude whether different muscles are affected in the same way. While both peripheral and ventilatory muscle dysfunction are deleterious, clinical studies in mechanically ventilated patients have demonstrated that their impact on patients' outcomes is different (Dres et al., 2017; Dres et al., 2019). Moreover, affection of distinct muscles could benefit from specific preventive and therapeutic approaches (Dong et al., 2021; Leite et al., 2018; Martin et al., 2011; Nakanishi et al., 2020; Nakano et al., 2021; Sotak et al., 2021).

Therefore, our study aimed to describe the morphologic and functional characteristics of different skeletal muscles during the course of acute lung injury (ALI). We used a previously established animal model of ALI developed by intratracheal instillation of lipopolysaccharides (LPS), which reproduces many characteristics of human ARDS. This model was chosen because it has already been used to study ALI‐related skeletal muscle compromise in hindlimb of mice (Files et al., 2012; Files et al., 2016). The model is characterized by a sublethal lung injury, with complete pulmonary recovery within 1 week. Hence, time points within this period were selected in order to describe the evolution of muscle affection through different stages of ALI. Furthermore, to comprehend the impact of ALI on different muscle types, all analyses were performed in a predominantly slow‐twitch and in a predominantly fast‐twitch hindlimb muscles, soleus and extensor digitorum longus (EDL), respectively; as well as in the diaphragm (predominantly fast‐twitch fibers) as the main respiratory muscle. We hypothesized that different profiles of atrophy and contractile dysfunction would be detected in respiratory, peripheral slow‐twitch and peripheral fast‐twitch muscles.

## 2 METHODS

### 2.1 Ethics statement

Experiments were performed following the ARRIVE guidelines, under a project license granted by the institutional ethics committee (approval no. 070153–000560‐14, Comisión de Ética en el Uso de Animales, Facultad de Medicina, Universidad de la República).

### 2.2 Animal model

Ten‐week‐old male C57BL/6 mice were provided by Facultad de Medicina and maintained under standard laboratory conditions (23°C, 12:12‐h light–dark cycle) with food and water accessible *at libitum*. Mice were anesthetized with isoflurane and intubated with a 20‐gauge catheter. LPS from *Escherichia coli* 055:B5 (L2880, Sigma‐Aldrich) at 3 μg/g of body weight was instilled intratracheally in order to induce lung injury (Files et al., 2012). Sham control animals received a similar volume of sterile saline instead of LPS. At different time points, animals were anesthetized with sodium pentobarbital (40 mg/kg intraperitoneal) for tissue sampling. Animals were euthanized by exsanguination. Sham animals were studied 3 days after saline instillation. For all experiments *n* ≥ 6 animals per group, unless otherwise specified.

### 2.3 Bronchoalveolar lavage and analysis

The bronchoalveolar lavage fluid (BALF) was obtained after instillation of 1 ml of sterile saline through an airway catheter. White blood cell (WBC) count in the BALF was determined using an automated hematology analyzer (Cell‐Dyn Ruby, Abbott Core Laboratory Systems). To measure protein concentration BALF was centrifuged at 200*g* for 5 min and the supernatant was analyzed with the bicinchoninic acid assay.

### 2.4 Lung histology

A 20‐gauge catheter was sutured into the trachea, the lungs were carefully excised and inflated to 15 cmH_2_O with 4% buffered formaldehyde. Lungs were embedded in paraffin and 4‐μm sections were stained with hematoxylin and eosin (H&E) for histologic evaluation (Optiphot; Nikon).

### 2.5 Muscle procurement and determination of muscle mass

At selected time points (2, 4, and 7 days after LPS administration, i.e. LPS‐d2‐4‐7) hindlimb and respiratory skeletal muscles were carefully dissected under real‐time magnification. Soleus and EDL were extracted preserving proximal and distal tendons. Subsequently, the diaphragm was dissected preserving the central tendon and the costal insertions. Muscle mass was determined for soleus and EDL after removing fat, tendons, and remaining blood using an analytical balance (ED124S, Sartorius). Muscle mass was normalized by tibia length.

### 2.6 Muscle histology

Muscles were fixed with 4% buffered formaldehyde and embedded in paraffin. Serial transverse cryosections (4 μm) were stained with H&E for histologic evaluation. Photomicrographs were taken at 40× magnification (Optiphot, Nikon, Japan). For each muscle fiber cross‐sectional area (CSA) was measured in ≥200 fibers (ImageJ, National Institutes of Health) and expressed as mean fiber CSA (μm^2^) and fiber size distribution (Jaitovich et al., 2015).

### 2.7 Muscle in vitro contractility

Muscle contractile properties were studied in vitro under isometric conditions in sham, LPS‐d3, and LPS‐d7 mice (Angulo et al., 2009). Muscles were surgically excised taking special care not to stretch or damage the fibers. Muscles were immediately placed in chilled (4°C) Krebs solution (in mM: NaCl 118, KCl 4.7, CaCl_2_ 2.5, MgSO_4_ 1.2, KH_2_PO_4_ 1, NaHCO_3_ 25, glucose 11; pH 7.4) bubbled with 95% O_2_–5% CO_2_ (Barreiro et al., 2002). A muscle strip (3–4 mm wide) of the lateral portion of the diaphragm was dissected, along with the orientation of the fibers, with part of the rib and central tendon attached for mounting. Soleus and EDL muscles were mounted through proximal and distal tendons. Muscles were transferred to a vertical organ bath (MyoBath, World Precision Instruments, Inc., Sarasota, FL) filled with equilibrated Krebs solution (95% O_2_–5% CO_2_) kept at 23°C, with a constant flow of approximately 10 ml/min. A 4–0 silk suture was used to secure each muscle to an isometric force transducer (FORT100, World Precision Instruments, Inc., Sarasota, FL) and allowed to equilibrate for 15 min. Muscles were subsequently stimulated via two platinum electrodes using 1‐ms square current pulses at supramaximal voltage (Grass S48 Stimulator, Grass Instruments). All experimental procedures were conducted maintaining optimal muscle fiber length (*L*_o_), defined as the muscle length at which maximal twitch tension was obtained. Force measurements were recorded and analyzed with AxoScope Software (Molecular Devices).

#### 2.7.1 Single twitch

Twitch contractile properties were analyzed by performing a single electrical pulse (1 Hz) and measuring: single twitch tension, contraction time to peak tension (CT), half relaxation time (HRT, time required for peak tension to decrease by 50%), contraction speed (dT/dt_max_, maximum rate of rising of peak tension) and relaxation speed (−dT/dt_max_, maximum rate of decrease in peak tension).

#### 2.7.2 Force‐frequency relationship

Maximal isometric tetanic tension was determined at different stimulating frequencies (10, 20, 30, 50, 80, 100, and 120 Hz, 1‐s train duration), with 1 min between each stimulation train.

#### 2.7.3 Fatigue resistance and recovery

To examine fatigue muscles were stimulated at 20 Hz, 500‐ms train duration, 1 train/s, for 5 min. Fatigue resistance was determined by comparing the tension generated on the first and last train (%). Immediately after the fatigue protocol, maximum tetanic stimuli (120 Hz, 1‐s train duration) were performed at subsequent time points (0, 1, 2, 3, 4, and 5 min) and fatigue recovery was determined by comparing the generated tension to pre‐fatigue 120 Hz tension.

At the end of the experiment muscle length at L_o_ was measured using a digital caliper. Muscle was freed from tendons and ribs and weighted. Absolute tension (g) generated during contraction was normalized for CSA and expressed as specific force (N/cm^2^) using the following formula: tension (kg) × 9.8 (gravitational constant, m/s^2^) × length (cm) × 1.056 (muscle density, g/cm^3^) / weight (g) (Supinski et al., 1999).

### 2.8 Western blot

Muscle samples were mechanically homogenized on ice with cold lysis buffer in a 10‐fold excess (wt/wt), containing NaF 5 mM, β‐glycerophosphate 50 mM, RIPA buffer (Tris pH 8 25 mM, NaCl 150 mM, NP‐40 1%, deoxycholate 1%, sodium dodecyl sulfate 0.1%) and protease inhibitors with a tissue homogenizer (T10 basic ULTRA‐TURRAX, Ika). Samples were centrifuged at 12,000*g* for 15 min at 4°C and the supernatant was collected. The Bradford assay was employed to measure protein concentration (Thermo Scientific Protein Biology Products). Proteins were separated by SDS‐PAGE and semi‐dry transferred to nitrocellulose membranes for immunoblotting. Primary antibodies for Pax‐7 (Santa Cruz Biotechnology Cat# sc‐81,975, RRID:AB_2252008) and GAPDH (Santa Cruz Biotechnology Cat# sc‐32,233, RRID:AB_627679) were used and results were visualized by chemiluminescence using horseradish peroxidase‐conjugated secondary antibodies according to the manufacturer's instructions (Thermo Scientific Protein Biology Products).

### 2.9 Statistical analysis

Data are expressed as mean (SD) or absolute frequency (%), unless otherwise specified. Comparisons between different groups were performed with one‐way ANOVA followed by Bonferroni post hoc test. Two‐way ANOVA and Bonferroni tests were used to analyze force‐frequency relationships and fatigue recovery. Results were considered statistically significant when *p* < 0.05. Prism 6.01 software (GraphPad) was used for the analysis.

## 3 RESULTS

### 3.1 Acute lung injury in mice

**FIGURE 1:**
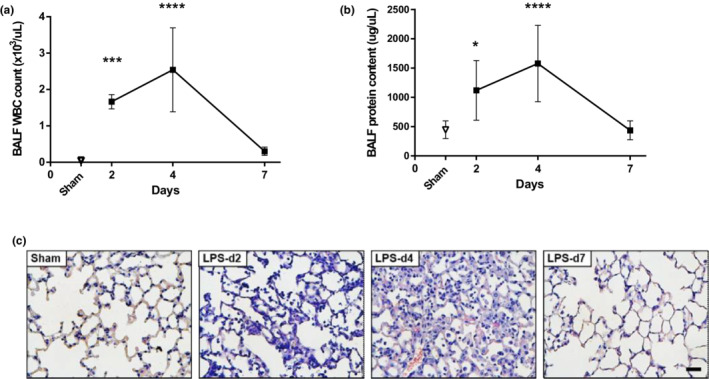
Evolution of lung injury after intratracheal LPS instillation. (a) white blood cell (WBC) count in bronchoalveolar lavage fluid (BALF). (b) protein concentration in BALF. (c) lung sections stained with hematoxylin and eosin. Scale bars = 20 μm. *p < 0.05, ***p < 0.001, ****p < 0.0001 compared to control animals (sham).

Lipopolysaccharide's instillation resulted in acute and reversible lung injury. A significant increase in protein concentration and WBC count in BALF was observed on days 2 and 4 after LPS administration, returning to baseline levels (sham group) by day 7 (Figure 1a,b). In concordance, cellular infiltrates and interstitial thickening was evident in histologic analysis on days 2 and 4, but resolved by day 7 after LPS instillation (Figure 1c).

### 3.2 Skeletal muscle atrophy

**FIGURE 2:**
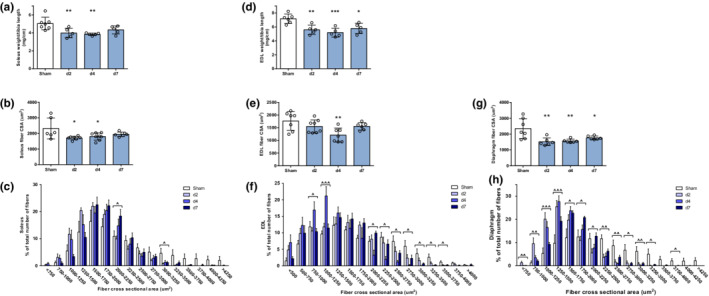
Skeletal muscle atrophy in mice with lung injury. (a) soleus muscle weight normalized by tibia length. (b) soleus muscle fibers' mean cross‐sectional area (CSA). (c) histogram of soleus fiber size distribution. (d) extensor digitorum longus (EDL) muscle weight normalized by tibia length. (e) EDL muscle fibers' mean CSA. (f) histogram of EDL fiber size distribution. (g) diaphragm muscle fibers' mean CSA. (h) histogram of diaphragm fiber size distribution. *p < 0.05, **p < 0.01, ***p < 0.001 compared to control animals (sham). ^*p* < 0.05, ^^*p* < 0.01, ^^^*p* < 0.001 between groups.

**FIGURE 3:**
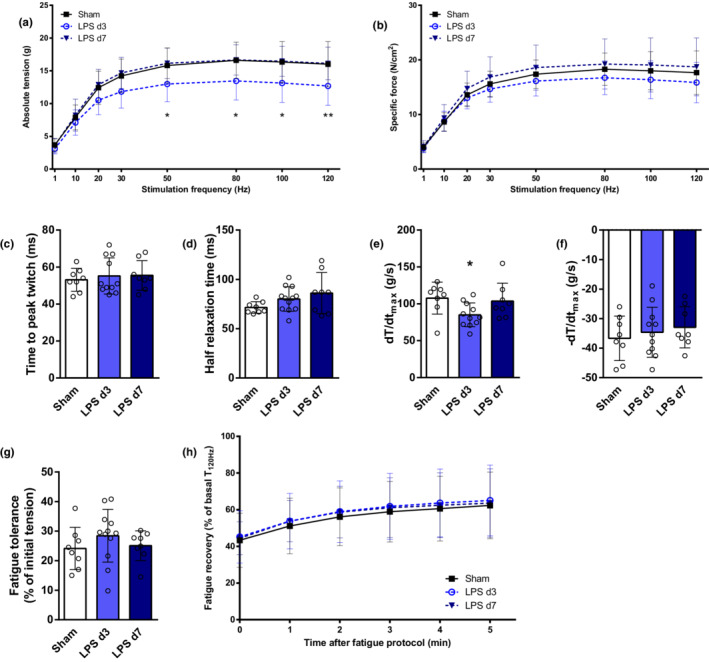
Soleus in vitro contractility analysis in control mice (sham), 3 days (LPS d3) and 7 days (LPS d7) after LPS instillation. Soleus absolute tension (a) and specific force (b) at different stimulation frequencies were recorded. Contraction time (c), half relaxation time (d), contraction speed (dT/dt_max_, e) and relaxation speed (−dT/dt_max_, f) were measured from a single twitch (1 Hz). Fatigue resistance (g) and recovery after fatigue (h) are presented. *p < 0.05, **p < 0.01 comparing sham versus LPS d3.

Atrophy was detected in the three study muscles after LPS administration. A significant decrease was observed in soleus wet weight and in the fibers' mean CSA by days 2 and 4, with a gradual recovery by day 7 (Figure 2a,b). Accordingly, a leftward shift in the fibers size distribution histogram occurred in animals treated with LPS (Figure 2c). Wet muscle weight of EDL was significantly reduced after LPS administration, even after lung injury resolved (day 7, Figure 2d). A decrease in EDL fibers' CSA (reaching a nadir at day 4) and a predominance of smaller fibers was also observed after LPS instillation (Figure 2e,f). Finally, a profound and persistent reduction in diaphragm fibers' CSA occurred in mice following LPS administration (Figure 3g,h).

### 3.3 Skeletal muscle contractility

Different patterns of muscle function compromise were observed in the three studied muscles. In soleus muscle, a significant reduction in absolute tension (from 50 to 120 Hz stimuli) was observed during the acute phase of lung injury (LPS‐d3), which was completely restored on day 7 (Figure 3a). Nevertheless, soleus specific force was not affected in mice with lung injury (Figure 3b). A significant decrease in dT/dt_max_ could be detected in mice on the third day of LPS instillation, without affection of another soleus' twitch contractile kinetics (Figure 3c–f). Soleus muscle fatigue resistance and recovery were not different between animal groups (Figure 3g,h).

**FIGURE 4:**
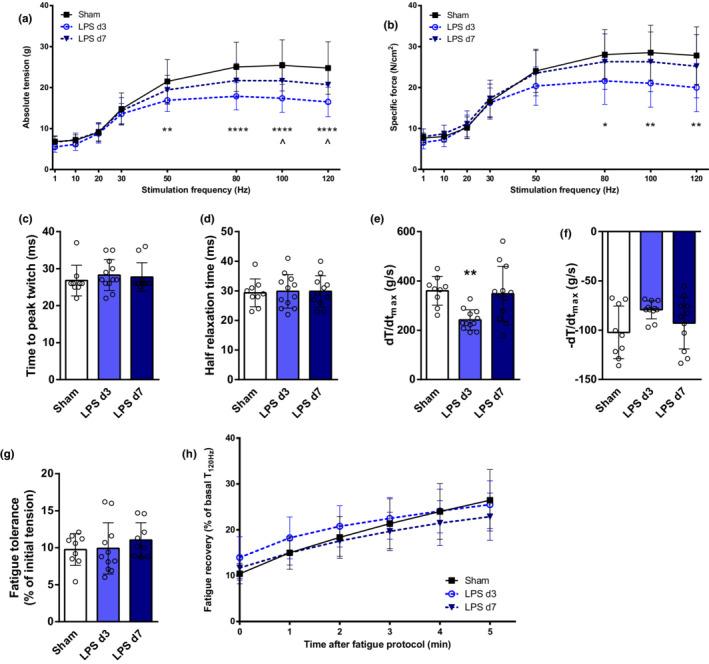
Extensor digitorum longus (EDL) in vitro contractility analysis in control mice (sham), 3 days (LPS d3) and 7 days (LPS d7) after LPS instillation. EDL absolute tension (a) and specific force (b) at different stimulation frequencies were recorded. Contraction time (c), half relaxation time (d), contraction speed (dT/dt_max_, e) and relaxation speed (−dT/dt_max_, f) were measured from a single twitch (1 Hz). Fatigue resistance (g) and recovery after fatigue (h) are presented. *p < 0.05, **p < 0.01, ****p < 0.0001 comparing sham versus LPS d3. ^*p* < 0.05 comparing sham versus LPS d7.

Contractile properties of EDL muscle were more compromised. A robust and significant decline was observed in EDL absolute tension in LPS‐d3 mice, which was not completely restored by day 7 (Figure 4a). Specific force was also significantly reduced in LPS‐d3, but returned to near normal (sham) levels in LPS‐d7 animals (Figure 4b). No differences were observed in EDL single twitch CT and HRT (Figure 4c,d). Contraction speed (dT/dt_max_) was significantly lower in EDL of LPS‐d3 mice, but similar to control in LPS‐d7 animals (Figure 4e). A not statistically significant reduction in relaxation speed (−dT/dt_max_) could be detected 3 days after LPS instillation (Figure 4f). No differences were observed in EDL muscle fatigue resistance and recovery among sham, LPS‐d3, and LPS‐d7 mice (Figure 4g,h).

**FIGURE 5:**
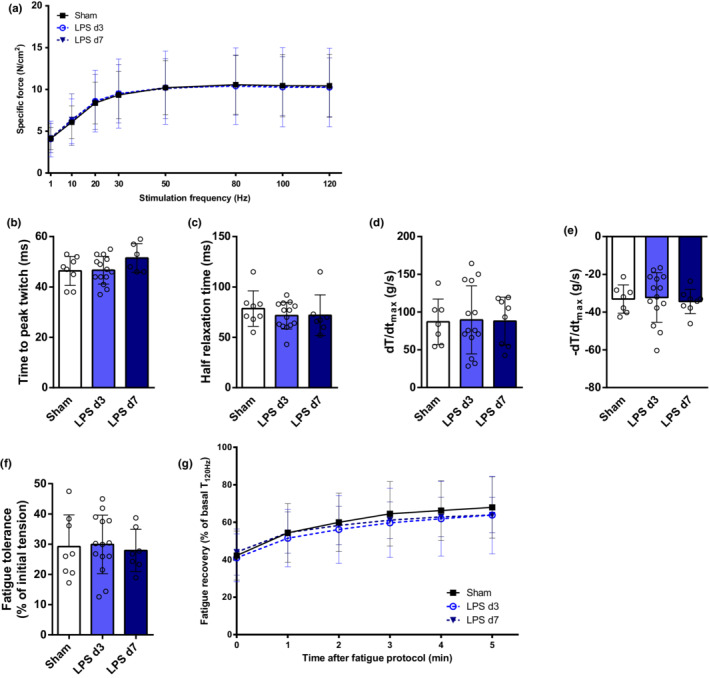
Diaphragm in vitro contractility analysis in control mice (sham), 3 days (LPS d3) and 7 days (LPS d7) after LPS instillation. Diaphragm specific force at different stimulation frequencies was recorded (a). Contraction time (b), half relaxation time (c), contraction speed (dT/dt_max_, d) and relaxation speed (−dT/dt_max_, e) were measured from a single twitch (1 Hz). Fatigue resistance (f) and recovery after fatigue (g) are presented.

Finally, all diaphragmatic contractile properties (single twitch kinetics, force‐frequency relationship, fatigue resistance, and fatigue recovery) were similar between sham, LPS‐d3, and LPS‐d7 animals (Figure 5).

### 3.4 Pax7 expression

**FIGURE 6:**
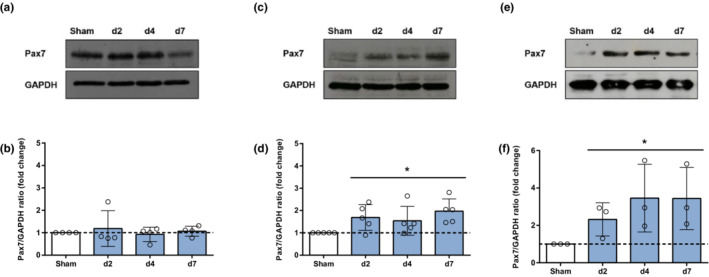
Pax7 protein expression in soleus, extensor digitorum longus (EDL), and diaphragm after LPS‐induced lung injury. Representative immunoblots and densitometry of Pax7 in soleus (a, b), EDL (c, d), and diaphragm (e, f) in control mice (sham) and at days 2, 4, and 7 after LPS instillation (*n* ≥ 3). GAPDH was used as loading control. Cropped blot images are presented for clarity. *p < 0.05 compared to sham.

Skeletal muscle recovery after injury or atrophy is mainly determined by the myogenic precursor satellite cell (Chen & Shan, 2019; Fukada, 2018). In order to get an initial insight regarding the myogenic response to acute lung injury, expression of the satellite cell‐specific transcription factor Pax7 was determined in the different muscles by immunoblotting. We did not find differences in soleus Pax7 expression after intratracheal LPS administration (Figure 6a,b). However, a remarkable and persistent increase in Pax7 protein expression was observed in EDL (1.5‐2.0‐fold, *p* < 0.05) and diaphragm (2.3–3.5‐fold, *p* < 0.05) of mice with lung injury (Figure 6c–f).

## 4 DISCUSSION

In the current study, the most relevant result was that different peripheral and respiratory skeletal muscles are distinctly affected during the course of ALI. In fact, each of the studied muscles presented a unique pattern in terms of atrophy development, contractile dysfunction and Pax7 expression. In the fast‐twitch hindlimb muscle EDL, persistent muscle wasting was observed, with a transient impairment of contractility, and a significant increase in Pax7 expression. In contrast, although atrophy was also detected in the slow‐twitch hindlimb muscle soleus, contractility was not affected, and Pax7 expression remained unchanged. Finally, profound muscle atrophy was observed in the diaphragm, with a remarkable increase in Pax7 expression but without contractile dysfunction.

### 4.1 Muscle atrophy in acute lung injury

Previous work from Files et al. demonstrated that muscle wasting observed in mice with ALI induced by intratracheal LPS instillation was mediated through the E3 ubiquitin ligase MuRF1 (Files et al., 2012). The authors described atrophy occurring at the hindlimb fast‐twitch muscle tibialis anterior (TA), as well as a decrease in hindlimb contractile force determined in vivo. The pattern of muscle compromise described both in patients with ARDS and in different animal models is highly heterogeneous (Carambula et al., 2021; Divangahi et al., 2004; Shiota et al., 2004). Therefore, we aimed to further investigate the morphologic and functional properties of three different muscles during the course of murine acute lung injury and resolution stages.

Muscle atrophy was developed in all three studied muscles after LPS instillation (Figure 2). Both hindlimb muscles were affected by muscle wasting. Slow‐twitch (oxidative) and fast‐twitch (glycolytic) fibers have different susceptibility to specific atrophy signals.(Wang & Pessin, 2013) Although differential fiber type CSA analysis was not performed in our study, a similar degree and evolutionary pattern of atrophy were observed in predominantly slow‐twitch (soleus) and fast‐twitch (EDL) muscles. Our experiments also demonstrated a more severe and persistent atrophy of the diaphragm, as compared to peripheral muscles. Interestingly, muscle mass (particularly diaphragmatic) was not completely restored by the time lung injury was resolved (day 7).

### 4.2 Muscle dysfunction in acute lung injury

Muscle force‐generation capacity depends on muscle mass, specific contractility, and neuromuscular transmission. In this animal model, a reduction in hindlimb dorsiflexion absolute force (mainly determined by TA contraction) (Gerlinger‐Romero et al., 2019) was previously reported, but without affection of specific force (Files et al., 2012). However, in our study different patterns of muscle function compromise were observed. Noteworthy, intrinsic contractile properties of EDL muscle were significantly affected. Therefore, the profound decline in EDL's absolute force observed in LPS‐d3 (Figure 4a) reflects muscle atrophy (Figure 2d–f) and impaired specific contractility (Figure 4b). However, while EDL's specific force was restored in LPS‐d7, absolute strength was still reduced because of persistent muscle atrophy. In contrast to EDL, soleus and diaphragm specific force were not affected during the course of lung injury (Figures 3b and 5a). Hence, the reduced soleus absolute force on LPS‐d3 (Figure 3a) is probably a consequence of muscle atrophy at that time point (Figure 2a–c). While the absolute force of the whole diaphragm could not be determined in vitro in this setting, the pronounced and persistent atrophy observed (Figure 3g,h) suggests that respiratory muscle capacity is probably reduced in mice treated with LPS.

The heterogeneous compromise of intrinsic contractile properties found in our study is particularly interesting. Specific force was impaired in EDL muscle during the phase of active lung injury, but preserved in soleus and diaphragm. Different muscle susceptibility to contractile dysfunction has been already described in numerous animal models. Chronic hypoxia in rats determined a severe reduction in EDL's contractility, with a minor attenuation on diaphragmatic force and no effect on soleus (Shiota et al., 2004). Hemorrhagic shock was associated with a dramatic impairment of soleus contractility but preserved diaphragmatic function (Carreira et al., 2014). On the contrary, *Pseudomonas aeruginosa* lung infection in mice caused a significant impairment of diaphragmatic contractility without affecting soleus or EDL (Divangahi et al., 2004). There is no clear explanation for these discrepancies among different animal models. Undoubtedly, fiber type composition (fast‐twitch/slow‐twitch), muscle group (peripheral/respiratory) and workload (disuse/overload) are major determinants of the way each muscle is affected in a particular scenario. However, other factors such as calcium homeostasis, myofibrillar composition, and mitochondrial function could also be decisive (Ottenheijm et al., 2008; Picard et al., 2012; van Hees et al., 2012).

### 4.3 Differential Pax7 expression in response to acute lung injury

Skeletal muscle has a remarkable capacity to regenerate upon injury, which is mainly accomplished by recruiting myogenic stem cells, called satellite cells (Mauro, 1961). The muscle regeneration process might be a key factor to better understand skeletal muscle compromise in patients recovering from ARDS. In fact, reduced content of satellite cells was observed in patients with sustained muscle atrophy after intensive care unit discharge (Dos Santos et al., 2016).

Upon activation, the transition of satellite cells through the different stages of myogenesis is regulated by the sequential expression of specific transcription factors (Guitart et al., 2018). Among them, Pax7 is considered the key biomarker of satellite cells. Pax7 is uniquely expressed by satellite cells, and its upregulation is essential and characteristic during satellite cell activation and proliferation (Seale et al., 2000). Therefore, the increased expression of Pax7 in diaphragm and EDL suggests that quiescent satellite cells are activated in response to LPS‐induced ALI in these muscles. However, more specific techniques would be required to confirm this presumption. Surprisingly, increased expression of Pax7 was not observed in soleus muscle despite presenting a similar degree of atrophy. Differences among fast‐twitch and slow‐twitch muscles in the regeneration process after distinct insults have already been described by other authors, and could be related to differences in muscle innervation, specific inflammatory response or intrinsic characteristics of particular satellite cells populations (Kalhovde et al., 2005; Zimowska et al., 2017). A thorough characterization of satellite cells' activation, proliferation, and differentiation process might help to fully elucidate how the muscle regeneration progress unfolds in each muscle; however, such an approach is beyond the scope of this paper.

### 4.4 Study limitations

Our study has certain limitations. First, it was conducted using a particular animal model in specific time points selected to focus on the acute and sub‐acute stages of lung injury (Files et al., 2012; Files et al., 2016). However, whether the results could be extrapolated to different models or time points is uncertain. Future research is required to assess the long‐term effects of ALI on skeletal muscles. Second, reduced food intake and mobility could have contributed to muscle wasting. While this makes it harder to determine the particular role of lung injury per se in skeletal muscle alterations, these factors are also commonly present in patients with ARDS (Reeves et al., 2014). However, a pair‐fed group would be required in order to determine if any of the observed alterations are related to reduced food intake. Additionally, mice were not exposed to factors that might contribute to muscle injury in ARDS patients, such as mechanical ventilation, asynchronies, neuromuscular blockers, etc. Third, although soleus and EDL were selected in order to represent slow‐twitch and fast‐twitch muscles respectively, differential fiber type CSA was not analyzed. A more detailed analysis of different fiber types is required to determine which fibers are compromised, and whether fiber type switch occurs in response to ALI. Finally, given the descriptive design of our work, further research should be conducted in order to uncover the underlying mechanisms of different skeletal muscles' compromise in ALI. Particularly, satellite cells response and myogenic process should be studied in detail through more precise methods.

#### CONCLUSIONS

Muscle compromise in response to ALI is heterogeneous in this animal model. While specific contractility was only transiently impaired in a single muscle type, muscle wasting was observed in all the studied muscles, persisting even after lung injury has resolved. However, myogenic response varied among muscles, as suggested by a differential expression of satellite cell‐specific transcription factor Pax7. A more profound description of the muscle regeneration process in ALI remains to be performed. This knowledge might help to optimize muscle rehabilitation in patients with ARDS.

## AUTHOR CONTRIBUTIONS

Martín Angulo: designed and conceived the study, obtained funding, performed experiments, analyzed results and wrote the manuscript; Agustina Vacca: performed experiments and analyzed results; Romina Rodríguez, María Noel Marin, Ana Laura Suárez, Gissel Jorge, Oscar Nosiglia, Victoria Cambón, Anaclara Ríos and Matías Iglesias: performed experiments and acquired data; Mariana Seija: supervised animal studies; Carlos Escande: provided reagents and equipment; Javier Hurtado and Arturo Briva: participated in study conception. All authors approved the final version of the manuscript and agree to be accountable for all aspects of the work in ensuring that questions related to the accuracy or integrity of any part of the work are appropriately investigated and resolved.

## FUNDING INFORMATION

This study was supported by Comisión Sectorial de Investigación Científica and Agencia Nacional de Investigación e Innovación.

## CONFLICT OF INTERESTS

None.
